# Combination of brain natriuretic peptide and urinary albumin as a predictor of cardiovascular–renal events in outpatients with chronic kidney disease

**DOI:** 10.20407/fmj.2022-004

**Published:** 2022-07-22

**Authors:** Shoya Oyama, Hiroshi Takahashi, Hiroki Hayashi, Shigehisa Koide, Shigeru Nakai, Kazuo Takahashi, Daijo Inaguma, Midori Hasegawa, Junichi Ishii, Yukio Yuzawa, Naotake Tsuboi

**Affiliations:** 1 Department of Nephrology, Fujita Health University, School of Medicine, Toyoake, Aichi, Japan; 2 Faculty of Clinical Engineering, Fujita Health University, School of Medical Sciences, Toyoake, Aichi, Japan; 3 Department of Biomedical Molecular Sciences, Fujita Health University, School of Medicine, Toyoake, Aichi, Japan; 4 Department of Internal Medicine, Fujita Health University Bantane Hospital, Nagoya, Aichi, Japan; 5 Department of Cardiovascular Medicine, Fujita Health University, School of Medicine, Toyoake, Aichi, Japan

**Keywords:** Brain natriuretic peptide, Urinary albumin, Cardiovascular–renal events, Chronic kidney disease

## Abstract

**Objectives:**

Cardiovascular and renal diseases are closely related. Brain natriuretic peptide (BNP) and urinary albumin are established predictors for cardiac and renal morbidities, respectively. To date, no reports have investigated the combined predictive value of BNP and urinary albumin for long-term cardiovascular–renal events in patients with chronic kidney disease (CKD). The aim of this study was to investigate this theme.

**Methods:**

Four hundred eighty-three patients with CKD were enrolled into this study and followed-up for 10 years. The endpoint was cardiovascular–renal events.

**Results:**

During the median follow-up period of 109 months, 221 patients developed cardiovascular–renal events. Log-transformed BNP and urinary albumin were identified as independent predictors for cardiovascular–renal events, with a hazard ratio of 2.59 (95% confidence interval [CI], 1.81–3.72) and 2.27 (95% CI, 1.82–2.84) for BNP and urinary albumin, respectively. For the combined variables, the group with high BNP and urinary albumin had a markedly higher risk (12.41-times; 95% CI 5.23–29.42) of cardiovascular–renal events compared with that of the group with low BNP and urinary albumin. Adding both variables to a predictive model with basic risk factors improved the C-index (0.767, 0.728 to 0.814, p=0.009), net reclassification improvement (0.497, p<0.0001), and integrated discrimination improvement (0.071, p<0.0001) more than each of them alone.

**Conclusions:**

This is the first report to demonstrate that the combination of BNP and urinary albumin can stratify and improve the predictability of long-term cardiovascular–renal events in CKD patients.

## Introduction

In patients with chronic kidney disease (CKD), a decrease in the glomerular filtration rate (GFR) and an increase in urinary albumin levels are associated independently with an increased risk of end-stage renal failure and cardiovascular death.^1,2^ Conversely, in patients with cardiac disease, renal function is impaired through hemodynamic involvement, such as decreased renal blood flow and/or venous congestion, and activation of the renin–angiotensin and sympathetic nervous systems.^3^ The umbrella term, cardiorenal syndrome (CRS), has been used to refer to a disorder of the heart and kidneys where acute or chronic dysfunction in one organ may induce acute or chronic dysfunction in another organ.

Biomarkers are helpful in assessing these conditions. Urinary albumin, a representative urinary biomarker, is related to cardiovascular disease (CVD) and death as well as to renal prognosis.^4^ Brain natriuretic peptide (BNP), a representative cardiac biomarker, was shown to be useful as a diagnostic, prognostic, and therapeutic indicator of heart failure (HF).^5^ To date, there have been no reports that investigate the combined predictive value of BNP and urinary albumin for long-term cardiovascular–renal events in patients with CKD. The aim of this study is to investigate this theme.

## Methods

### Study participants

Patients were selected from the cohorts that were enrolled in our previous studies^6–8^ between February 2009 and September 2010. The inclusion criteria were as follows: (1) urinary albumin >30 mg/g Cr or estimated GFR (eGFR) <60 mL/min/1.73 m^2^; (2) eGFR >15 mL/min/1.73 m^2^; (3) a follow-up period of at least 1 year; and (4) BNP and urinary albumin were measured at enrollment.

### Outcome assessment

The study participants were followed-up clinically until April 2020. The primary endpoint was the composite of the occurrence of cardiovascular events and progression of renal dysfunction. Cardiovascular events were defined as HF requiring hospitalization, myocardial infarction, angina pectoris requiring a therapeutic intervention, stroke, aortic dissection, or aortic aneurysm rupture. The Framingham criteria for HF^9^ was used. Stroke was defined as the presence of clinical signs of focal or global disturbances in cerebral function caused by cerebrovascular damage. Progression of renal dysfunction was defined as the doubling of serum creatinine (Cr) or the initiation of renal replacement therapy.

### Ethics

All procedures that involved human participants were performed in accordance with the ethical standards of the institutional and/or national research committee and with the 1964 Declaration of Helsinki and its later amendments or comparable ethical standards. This study was approved by the Ethics Committee at the Fujita Health University School of Medicine (authorized number: HM20-059). Informed consent was waived because of the retrospective nature of the study.

### Statistical analyses

Normally distributed variables are expressed as the mean±standard deviation, whereas nonparametric data are presented as the median and interquartile range. Intergroup differences were evaluated using a one-way analysis of variance, and the Kruskal–Wallis and Chi-square tests were used for continuous and categorical variables, respectively. Cumulative incidence rates of cardiovascular–renal events were estimated using the Kaplan–Meier method, and intergroup differences were compared using a log-rank test. BNP levels were divided into low (<40 pg/mL), moderate (40–100 pg/mL), and high (>100 pg/mL) categories, in accordance with the JCS 2017/JHFS 2017 Guideline on Diagnosis and Treatment of Acute and Chronic Heart Failure.^10^ Urinary albumin levels were divided into normal to mild (30 mg/g Cr), moderate (30–300 mg/g Cr), and high categories (>300 mg/g Cr) in accordance with the Kidney Disease Improving Global Outcomes (KDIGO) CKD guidelines.^2^ Hazard ratios (HRs) and 95% confidence intervals (CIs) for cardiovascular–renal events, cardiovascular events, or renal events were calculated for each factor using the Cox proportional hazards analysis after adjusting for age, sex, systolic blood pressure, hemoglobin, high-density lipoprotein (HDL)-cholesterol, low-density lipoprotein (LDL)-cholesterol, eGFR, history of CVD, and diabetes. To assess whether the accuracy of predicting endpoints would improve after combining BNP and urinary albumin in a baseline model using the already known risk^11–13^ score, we calculated the C-index, net reclassification improvement (NRI), and integrated discrimination improvement (IDI). The C-index, which is defined as the area under the receiver operating characteristic curve between the individual predictive probabilities for endpoints and the incidence of endpoints, was compared using the baseline model.^14^ The NRI is a relative indicator of how many patients showed an improvement in the predicted probability for the endpoints. The IDI indicates the average improvement in the predicted probability for the endpoints after adding variables to the baseline model.^15^ A p-value <0.05 was considered statistically significant. Statistical analyses were performed using SPSS version 25.0 (IBM Corp., Armonk, NY, USA).

## Results

### Baseline characteristics

**Figure 1:**
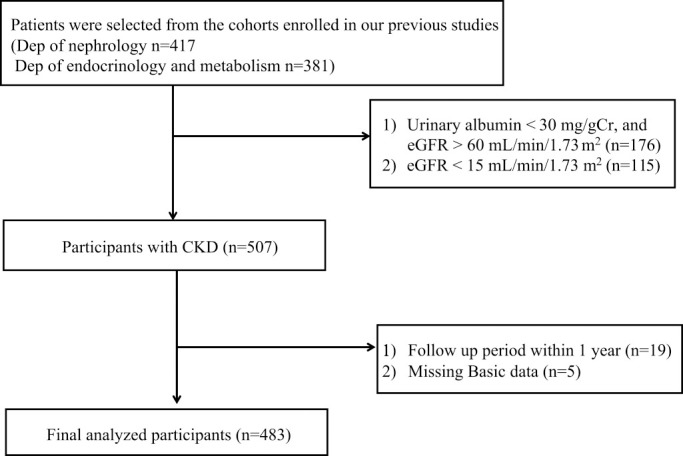
Flowchart of patient selection

Patient selection is shown in Figure 1, and 483 patients were enrolled. Study population characteristics are summarized in Table 1. The mean age was 67±13 years, and the prevalence of male sex, diabetes, smoking, and history of CVD was 62.7%, 57.8%, 32.1%, and 18.0%, respectively. Systolic and diastolic blood pressure was 130 (122–140) mmHg and 71 (67–78) mmHg, respectively. The eGFR was 40.2 (26.9–57.1) mL/min/1.73 m^2^. Clinical characteristics on the basis of the BNP category are shown in Table 2, and those on the basis of the urinary albumin category are shown in Table 3. The most common primary disease was diabetic nephropathy followed by nephrosclerosis and primary glomerular disease (Table 4).

### Correlated factors for brain natriuretic peptide and urinary albumin

Table 5 shows factors that were correlated with BNP and urinary albumin using multiple regression analysis. BNP correlated significantly with age, hemoglobin, history of CVD, and urinary albumin, whereas urinary albumin correlated significantly with age, sex, systolic blood pressure, hemoglobin, LDL cholesterol, eGFR, diabetes, and BNP. The standardization coefficient β for log BNP to log urinary albumin was 0.16 (p=0.0003), while that of log urinary albumin to log BNP was 0.18 (p=0.0003).

### Prognostic value of BNP and urinary albumin

During the follow-up period of 109 (56–120) months, 221 patients (45.8%) developed cardiovascular and renal events; these included doubling of serum Cr levels in 144 patients (29.8%), induction of dialysis in 101 patients (20.9%), HF in 42 patients (8.9%), angina pectoris/myocardial infarction in 41 patients (8.5%), cerebral stroke in 48 patients (9.9%), and aortic dissection/aneurysm rupture in four patients (0.8%). On Cox multivariable analysis, log BNP (adjusted HR [aHR], 2.59; 95% CI, 1.81–3.72; p<0.0001) and log urinary albumin (aHR, 2.27; 95% CI, 1.82–2.84; p<0.0001) were identified as independent predictors for cardiovascular–renal events after adjusting for sex, age, systolic blood pressure, hemoglobin, HDL-cholesterol, LDL-cholesterol, eGFR, history of CVD, and diabetes (Table 6). For cardiovascular events alone, log BNP (aHR, 3.12; 95% CI, 2.00–4.87; p<0.0001) and log urinary albumin (aHR, 1.67; 95% CI, 1.27–2.20; p=0.0002) were also independent predictors (Table 6). For renal events alone, similar results were obtained, as follows: log BNP (aHR, 1.70; 95% CI, 1.10–2.62; p=0.017) and log urinary albumin (aHR, 3.76; 95% CI, 2.77–5.12; p<0.0001) (Table 6).

**Figure 2:**
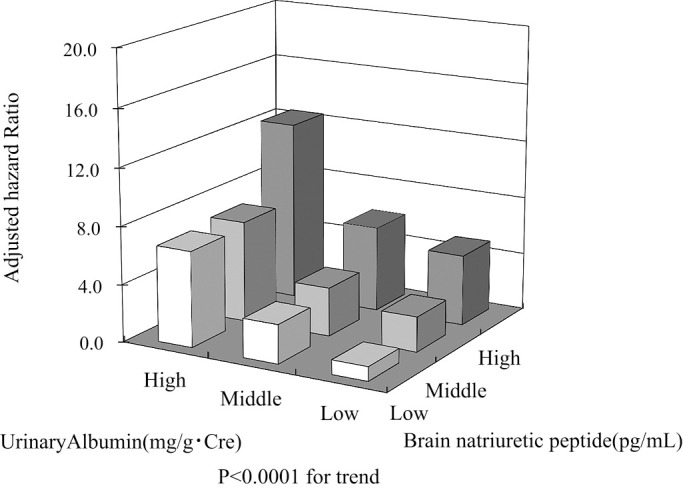
The cumulative incidence of cardiovascular–renal events over 10 years was 38.6%, 50.4%, and 63.4% in the low, moderate, and high BNP groups and 22.9%, 36.7%, and 67.0% in the low, moderate, and high urinary albumin groups, respectively (both p<0.0001).

**Figure 3:**
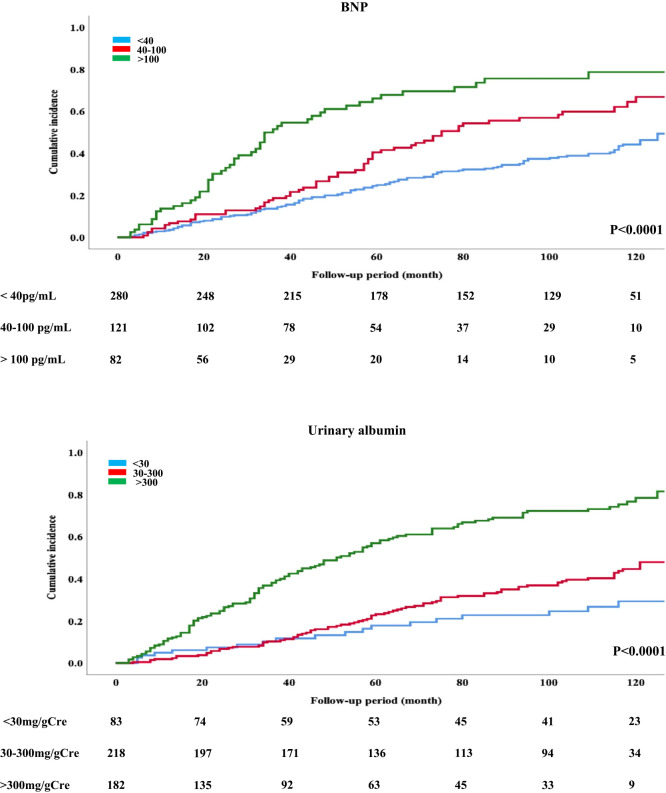
The hazard ratios for the occurrence of cardiovascular–renal events were stratified significantly into nine categories comprising combinations of the three brain natriuretic peptide categories and the three urinary albumin creatinine ratio categories.

When patients were divided into high, moderate, and low groups on the basis of BNP and urinary albumin levels, the high BNP group had a risk of cardiovascular–renal events that was 2.41-times (95% CI, 1.63–3.55; p<0.0001) higher than that of the low BNP group. A similar result was obtained on the basis of urinary albumin (aHR, 4.52; 95% CI, 2.69–7.60; p<0.001; Table 7). When both variables were combined, the group with high BNP and urinary albumin had a markedly higher risk (12.41-times; 95% CI 5.23–29.42, p<0.0001 for trend) of cardiovascular–renal events compared with that of the group with low BNP and urinary albumin (Table 8, Figure 2). The cumulative incidence of cardiovascular–renal events over 10 years was 38.6%, 50.4%, and 63.4% in the low, moderate, and high BNP groups and 22.9%, 36.7%, and 67.0% in the low, moderate, and high urinary albumin groups, respectively (both *P*<0.0001; Figure 3). Patient follow-up rates at 100 and 110 months were 76.4% and 72.5%, respectively.

### Identifying the predictive value for basic risk factors and the combination of BNP and urinary albumin for cardiovascular–renal events

We assessed the effect of adding BNP and urinary albumin to the basic risk factors comprising sex, age, systolic blood pressure, HDL-cholesterol, LDL-cholesterol, hemoglobin, eGFR, history of CVD, and diabetes.^11–13^ We found that adding BNP and urinary albumin improved the predictability for cardiovascular–renal events after follow-up for 109 months beyond that using the basic risk factors alone, as follows: C-index, 0.767 (95% CI, 0.728–0.814; p=0.009); NRI, 0.497 (p<0.0001); and IDI, 0.071 (p<0.0001). When BNP and urinary albumin are compared individually, the combination of BNP and urinary albumin significantly improved the NRI (0.460, p<0.0001 and 0.177, p=0.03, respectively) and IDI (0.053, p<0.0001 and 0.010, p=0.019, respectively; Table 9).

## Discussion

There were several main findings in this study. First, plasma BNP and urinary albumin levels, which are established markers for cardiovascular and renal disorders, respectively, were independently correlated with each other. Second, plasma BNP levels could predict both cardiovascular and renal events, and urinary albumin levels could predict renal and cardiovascular events, as previously reported. Third, the combination of both variables could stratify the risk of cardiovascular–renal events and improve the predictability (as shown by the increase in C-statistics, NRI, and IDI) better than each of the variables alone. Thus, these results might increase the knowledge about biomarkers for cardio-renal syndrome.

BNP is released by the left ventricle in response to left ventricular end-diastolic wall stress,^16^ and it is routinely used in clinical practice to diagnose or to exclude HF. BNP was classified as a Class 1 recommendation for diagnosis, severity, and prognostic evaluation in the 2017 guideline for diagnosis and treatment of acute and chronic HF.^10^

Patients with CKD have higher baseline BNP levels compared with those of matched patients with normal renal function because of impaired renal clearance, chronic pressure/volume overload, and CKD-associated cardiomyopathy.^17,18^ The following three fates are proposed for secreted BNP: (i) capture by its receptors for signal transduction or clearance; (ii) inactivation by neutral endopeptidase; or (iii) excretion from the kidney in its active form.^19^ In the highest urinary albumin category (300 mg/g Cr) in this study, the aHRs of cardiovascular–renal events between the BNP <40 pg/mL and 40<BNP<100 pg/mL categories were comparable. These results might be attributed to decreased excretion from the kidney that is caused by reduced renal function.

BNP levels are also significantly elevated in patients with CRS.^20^ In the consensus conference on CRS that was sponsored by the Acute Dialysis Quality Initiative (ADQI), CRS was classified into five subtypes.^3^ In CRS type 2, chronic HF results in CKD onset or progression. Examples of CRS type 2 include progressive CKD resulting from chronic HF in congenital or acquired heart disease or from repeated bouts of acute decompensated HF. Extended periods of chronic HF result in altered renal hemodynamics and progressive renal pathology related to albuminuria. The correlation of BNP with renal events in the present study may reflect this pathophysiology.

However, in CRS type 4, CKD promotes the progression of chronic HF, ventricular hypertrophy, and diastolic dysfunction and increased the risk of adverse cardiovascular events.^3^ The common traditional risk factors for CKD and CVD are older age, smoking, hypertension, diabetes, and dyslipidemia.^21^ Other factors associated with CKD, such as anemia, albuminuria, CKD–mineral and bone disorder, inflammation, increased renin–angiotensin–aldosterone system activation, sympathetic nervous system activation, oxidative stress, and insulin resistance, are also associated with the development of CVD.^13^ The correlation of urinary albumin with BNP also reflects these common backgrounds.

We assessed the effect of adding BNP and urinary albumin to the basic risk factors,^11–13^ which comprised age, sex, systolic blood pressure, hemoglobin, HDL-cholesterol, LDL-cholesterol, eGFR, history of CVD, and diabetes, and found that combining BNP and urinary albumin, rather than each factor alone, significantly improved the prediction of cardiovascular and renal events at 109 months better than the basic risk factors, as shown by the increase in the C-index, NRI, and IDI. Thus, measurement of both BNP and urinary albumin might improve the quality of risk management in clinical practice.

A meta-analysis showed a decrease in the hospitalization rate due to HF, CVD, or all-cause mortality when patients underwent BNP-guided therapy.^22^ A drug-induced reduction in urinary albumin was also found to be renoprotective.^23^ On the basis of the indications for drugs that have been shown to inhibit both cardiovascular and renal events, evaluating BNP combined with albuminuria might help to improve patients’ prognosis. Monitoring the change in BNP and albuminuria will also help to evaluate the effect of drugs such as renin–angiotensin–aldosterone system inhibitors^24^ and sodium–glucose cotransporter-2 inhibitors.^25^

Several reports have been published about the relationship between BNP and urinary albumin. Furukawa et al.^26^ researched the association between plasma BNP level and renal function among Japanese patients with type 2 diabetes mellitus. In their report, the highest BNP level (≥39.2 ng/mL) was independently and positively associated with microalbuminuria and macroalbuminuria. Naganuma et al.^27^ investigated BNP and urinary albumin as markers for cardiovascular evaluation of kidney donors. They found that plasma BNP concentrations in kidney donors were significantly higher than those in the healthy volunteers, and among kidney donors, 16% had microalbuminuria and 4% had overt proteinuria. Wang et al.^28^ evaluated the incremental usefulness of ten multiple biomarkers for predicting the risk of cardiovascular events in Framingham Heart Study participants. They showed that BNP and the urinary albumin-to-creatinine ratio were the best predicted major cardiovascular events. However, there have been no reports on the significance of the combination of BNP and urinary albumin as a predictor of cardiovascular–renal events over a long period in CKD patients. Thus, the present study is novel.

This study had some limitations. First, this was a single-center cohort study. Second, serum Cr levels were affected by both the eGFR and muscle mass. Third, we did not assess the serial changes in BNP and albuminuria over time.

In conclusion, BNP and urinary albumin are closely associated with long-term cardiovascular–renal events in CKD patients. The present report is the first to demonstrate that the combination of BNP and urinary albumin can stratify and improve the predictability of long-term cardiovascular–renal events in CKD patients.

## Figures and Tables

**Table1:** Patient demographic information (n=483)

	All patients (n=483)
Male sex (%)	62.7
Age (years)	67±13
Hypertension (%)	74.1
Diabetes (%)	57.8
Hyperlipemia (%)	41.4
Smoking (%)	32.1
History of cardiovascular disease (%)	18.0
Old myocardial infarction (%)	5.8
Heart failure (%)	6.6
Stroke (%)	5.6
Systolic blood pressure (mmHg)	130 (122–140)
Diastolic blood pressure (mmHg)	71 (67–78)
Albumin (g/dL)	4.1±0.5
Hemoglobin (g/dL)	12.5±2.0
HDL-cholesterol (mg/dL)	57±18
LDL-cholesterol (mg/dL)	113±33
eGFR (mL/min/1.73 m^2^)	40.2 (26.9–57.1)
hsCRP (mg/dL)	0.07 (0.03–0.17)
BNP (pg/mL)	31 (16–72)
Urinary albumin (mg/g Cr)	176 (46–601)
ARB (%)	66.0
ACEI (%)	18.2
β-blocker (%)	17.2
CCB (%)	53.2
Statins (%)	39.5
Aspirin (%)	22.2

HDL, high-density lipoprotein; LDL, low-density lipoprotein; eGFR, estimated glomerular filtration rate; hsCRP, high-sensitive C-reactive protein; BNP, brain natriuretic peptide; ARB, angiotensin receptor blocker; ACEI, angiotensin-converting enzyme inhibitor; CCB, calcium channel blocker

**Table2:** Clinical characteristics in accordance with brain natriuretic peptide category (n=483)

	BNP			p value
	Low (<40 pg/mL) (n=280)	Middle (40–100 pg/mL) (n=121)	High (>100 pg/mL) (n=82)	
Male sex (%)	63.6	61.2	62.2	0.90
Age (years)	63±13	71±10	73±10	<0.0001
Hypertension (%)	72.5	73.6	80.5	0.34
Diabetes (%)	60.4	57.9	48.8	0.18
Hyperlipemia (%)	39.6	42.1	46.3	0.71
Smoking (%)	33.6	29.8	30.5	0.49
History of cardiovascular disease				
Old myocardial infarction (%)	3.2	11.6	6.1	0.004
Heart failure (%)	1.8	5.8	24.4	<0.0001
Stroke (%)	6.1	5.0	4.9	0.85
Systolic blood pressure (mmHg)	129 (121–136)	131 (124–143)	131 (120–42)	0.046
Diastolic blood pressure (mmHg)	72 (68–78)	71 (65–78)	70 (64–78)	0.38
Albumin (g/dL)	4.2±0.4	4.0±0.5	3.8±0.5	<0.0001
Hemoglobin (g/dL)	13.0±1.8	11.9±2.1	11.4±1.9	<0.0001
HDL-cholesterol (mg/dL)	57±19	55±16	58±17	0.42
LDL-cholesterol (mg/dL)	117±32	110±32	105±34	0.009
eGFR (mL/min/1.73 m^2^)	43.9 (29.9–62.9)	37.3 (24.0–52.6)	32.4 (25.0–43.6)	<0.0001
hsCRP (mg/dL)	0.06 (0.02–0.16)	0.07 (0.03–0.17)	0.11 (0.04–0.32)	0.02
BNP (pg/mL)	19 (11–27)	60 (50–80)	172 (123–264)	<0.0001
Urinary albumin (mg/g Cr)	146 (39–573)	183 (57–560)	254 (51–1110)	0.10
ARB (%)	65.0	67.8	67.1	0.80
ACEI (%)	15.7	23.1	19.5	0.19
β-blocker (%)	10.0	23.1	32.9	<0.0001
CCB (%)	46.8	59.5	65.9	0.002
Statins (%)	43.2	32.2	37.8	0.12
Aspirin (%)	13.6	30.6	39.0	<0.0001

HDL, high-density lipoprotein; LDL, low-density lipoprotein; eGFR, estimated glomerular filtration rate; hsCRP, high-sensitive C-reactive protein; BNP, brain natriuretic peptide; ARB, angiotensin receptor blocker; ACEI, angiotensin-converting enzyme inhibitor; CCB, calcium channel blocker

**Table3:** Clinical characteristics on the basis of urinary albumin category (n=483)

	Urinary albumin			p value
	Low (<30 mg/g Cr) (n=83)	Middle (30–300 mg/g Cr) (n=218)	High (>300 mg/g Cr) (n=182)	
Male sex (%)	57.8	58.3	70.3	0.03
Age (years)	70±9	67±12	64±14	0.0006
Hypertension (%)	73.5	68.8	80.8	0.02
Diabetes (%)	45.8	62.4	57.7	0.03
Hyperlipemia (%)	32.5	40.8	46.2	0.10
Smoking (%)	27.7	29.8	36.8	0.07
History of cardiovascular disease				
Old myocardial infarction (%)	4.8	7.3	4.4	0.42
Heart failure (%)	8.4	3.7	9.3	0.05
Stroke (%)	7.2	6.0	4.4	0.64
Systolic blood pressure (mmHg)	125 (119–132)	130 (121–136)	132 (124–146)	<0.0001
Diastolic blood pressure (mmHg)	70 (64–76)	72 (68–78)	72 (67–79)	0.15
Albumin (g/dL)	4.1±0.4	4.2±0.4	3.9±0.5	<0.0001
Hemoglobin (g/dL)	12.7±2.0	12.8±1.9	12.0±2.1	0.0003
HDL-cholesterol (mg/dL)	57±20	58±18	55±16	0.23
LDL-cholesterol (mg/dL)	109±30	110±29	118±37	0.04
eGFR (mL/min/1.73 m^2^)	45.1 (31.8–53.8)	46.9 (34.0–71.6)	29.9 (22.2–45.4)	<0.0001
hsCRP (mg/dL)	0.06 (0.03–0.27)	0.08 (0.03–0.17)	0.07 (0.02–0.19)	0.51
BNP (pg/mL)	31 (18–64)	29 (15–61)	30 (19–87)	0.16
Urinary albumin (mg/g Cr)	13 (7–22)	89 (51–186)	934 (530–1955)	<0.0001
ARB (%)	65.1	59.6	74.2	0.006
ACEI (%)	13.3	16.5	22.5	0.12
β-blocker (%)	19.3	17.0	16.5	0.87
CCB (%)	37.3	48.6	65.9	<0.0001
Statins (%)	36.1	38.5	42.3	0.54

HDL, high-density lipoprotein; LDL, low-density lipoprotein; eGFR, estimated glomerular filtration rate; hsCRP, high-sensitive C-reactive protein; BNP, brain natriuretic peptide; ARB, angiotensin receptor blocker; ACEI, angiotensin-converting enzyme inhibitor; CCB, calcium channel blocker

**Table4:** Primary disease (n=483)

Primary disease	N (%)
Diabetic nephropathy	172 (35.6)
Nephrosclerosis	102 (21.1)
Primary glomerular disease	67 (13.9)
Vasculitis syndrome	20 (4.1)
Lupus nephritis	14 (2.9)
Tubular interstitial disease	11 (2.3)
Autosomal dominant polycystic kidney disease	6 (1.2)
Vascular disease other than vasculitis	4 (0.8)
Others	5 (1.0)
Unknown	82 (17.0)

**Table5:** Correlated factors for brain natriuretic peptide and urinary albumin by multiple regression analysis

	Standardization coefficient β	p value
log BNP		
Age	0.37	<0.0001
Sex (Male)	–0.05	0.24
Systolic blood pressure	–0.004	0.92
Hemoglobin	–0.20	0.0001
HDL-cholesterol	0.04	0.36
LDL-cholesterol	–0.08	0.07
eGFR	–0.02	0.66
History of cardiovascular disease	0.18	<0.0001
Diabetes	–0.01	0.81
log urinary albumin	0.16	0.0003

log urinary albumin		
Age	–0.31	<0.0001
Sex (Male)	0.17	0.0004
Systolic blood pressure	0.21	<0.0001
Hemoglobin	–0.18	0.001
HDL-cholesterol	–0.03	0.56
LDL-cholesterol	0.16	0.0002
eGFR	–0.22	<0.0001
History of cardiovascular disease	–0.05	0.29
Diabetes	0.14	0.002
log BNP	0.18	0.0003

HDL, high-density lipoprotein; LDL, low-density lipoprotein; eGFR, estimated glomerular filtration rate; BNP, brain natriuretic peptide

**Table6:** Predictive value of brain natriuretic peptide and urinary albumin for cardiovascular–renal events, cardiovascular events, and renal events

	HR (95%CI)	p value
Cardiovascular–renal events		
logBNP	2.59 (1.81–3.72)	<0.0001
log urinary albumin	2.27 (1.82–2.84)	<0.0001

Cardiovascular events		
logBNP	3.12 (2.00–4.87)	<0.0001
log urinary albumin	1.67 (1.27–2.20)	0.0002

Renal events		
logBNP	1.70 (1.10–2.62)	0.017
log urinary albumin	3.76 (2.77–5.12)	<0.0001

Adjusted for sex, age, systolic blood pressure, hemoglobin, HDL-cholesterol, LDL-cholesterol, eGFR, history of cardiovascular disease, and diabetes HR, hazard ratio; CI, confidence interval; HDL, high-density lipoprotein; LDL, low-density lipoprotein; eGFR, estimated glomerular filtration rate; BNP, brain natriuretic peptide

**Table7:** Adjusted hazard ratio of cardiovascular–renal events for brain natriuretic peptide and urinary albumin

		HR (95%CI)	p value
BNP	<40 pg/mL	Reference	
	40–100 pg/mL	1.24 (0.88–1.76)	0.22
	>100 pg/mL	2.41 (1.63–3.55)	<0.0001

Urinary albumin	<30 mg/g Cr	Reference	
	30–300 mg/g Cr	1.91 (1.14–3.21)	0.015
	>300 mg/g Cr	4.52 (2.69–7.60)	<0.0001

Adjusted for sex, age, systolic blood pressure, hemoglobin, HDL-cholesterol, LDL-cholesterol, eGFR, history of cardiovascular disease, and diabetes HDL, high-density lipoprotein; LDL, low-density lipoprotein; eGFR, estimated glomerular filtration rate; BNP, brain natriuretic peptide

**Table8:** Adjusted hazard ratio of cardiovascular–renal events in the combination of brain natriuretic peptide and urinary albumin

		Urinary albumin		
		<30 mg/g Cr	30–300 mg/g Cr	>300 mg/g Cr
BNP	<40 pg/mL	reference	2.70 (1.20–6.08)	6.65 (2.99–14.80)
	40–100 pg/mL	2.43 (0.76–7.79)	3.37 (1.44–7.88)	7.00 (2.99–16.39)
	>100 pg/mL	4.89 (1.62–14.79)	5.92 (2.35–14.88)	12.41 (5.23–29.42) (p<0.0001)

Adjusted for sex, age, systolic blood pressure, hemoglobin, HDL-cholesterol, LDL-cholesterol, eGFR, history of cardiovascular disease, and diabetes HDL, high-density lipoprotein; LDL, low-density lipoprotein; eGFR, estimated glomerular filtration rate; BNP, brain natriuretic peptide

**Table9:** Cardiovascular–renal events identified by each model using C-index, NRI, and IDI

	C-index (95%CI)	p value	NRI	p value	IDI	p value
Established risk factors^a^	0.727 (0.681–0.774)					
+BNP	0.739 (0.694–0.784)	0.19	0.267	0.002	0.018	0.002
+urinary albumin	0.766 (0.722–0.809)	0.009	0.449	<0.0001	0.061	<0.0001
+BNP+urinary albumin	0.767 (0.728–0.814)	0.009	0.497	<0.0001	0.071	<0.0001
+BNP vs. +BNP+urinary albumin	0.032 (0.006–0.058)^b^	0.014	0.460	<0.0001	0.053	<0.0001
+urinary albumin vs. +BNP+urinary albumin	0.006 (–0.006–0.017)^b^	0.33	0.177	0.03	0.010	0.019

^a^, adjusted for sex, age, systolic blood pressure, hemoglobin, HDL-cholesterol, LDL-cholesterol, eGFR, history of cardiovascular disease, and diabetes ^b^, estimated difference between two groups IDI, integrated discrimination improvement; NRI, net reclassification improvement; CI, confidence interval; HDL, high-density lipoprotein; LDL, low-density lipoprotein; eGFR, estimated glomerular filtration rate; BNP, brain natriuretic peptide

## References

[1] Herzog CA, Asinger RW, Berger AK, Charytan DM, Díez J, Hart RG, Eckardt KU, Kasiske BL, McCullough PA, Passman RS, DeLoach SS, Pun PH, Ritz E. Cardiovascular disease in chronic kidney disease. A clinical update from Kidney Disease: Improving Global Outcomes (KDIGO). Kidney Int 2011; 80: 572–586. 21750584 10.1038/ki.2011.223

[2] Stevens PE, Levin A. Evaluation and management of chronic kidney disease: synopsis of the kidney disease: improving global outcomes 2012 clinical practice guideline. Ann Intern Med 2013; 158: 825–830. 23732715 10.7326/0003-4819-158-11-201306040-00007

[3] Ronco C, McCullough P, Anker SD, et al. Cardio-renal syndromes: report from the consensus conference of the acute dialysis quality initiative. Eur Heart J 2010; 31: 703–711. 20037146 10.1093/eurheartj/ehp507 PMC2838681

[4] Viazzi F, Muiesan ML, Schillaci G, Salvetti M, Pucci G, Bonino B, Signori A, Pontremoli R. Changes in albuminuria and cardiovascular risk under antihypertensive treatment: a systematic review and meta-regression analysis. J Hypertens 2016; 34: 1689–1697. 27254313 10.1097/HJH.0000000000000991

[5] Nishikimi T, Kuwahara K, Nakao K. Current biochemistry, molecular biology, and clinical relevance of natriuretic peptides. J Cardiol 2011; 57: 131–140. 21296556 10.1016/j.jjcc.2011.01.002

[6] Maeda Y, Suzuki A, Ishii J, et al. Level of urinary liver-type fatty acid-binding protein is associated with cardiac markers and electrocardiographic abnormalities in type-2 diabetes with chronic kidney disease stage G1 and G2. Heart Vessels 2015; 30: 362–368. 24626813 10.1007/s00380-014-0489-4

[7] Hasegawa M, Ishii J, Kitagawa F, Kanayama K, Takahashi H, Ozaki Y, Yuzawa Y. Prognostic value of highly sensitive troponin T on cardiac events in patients with chronic kidney disease not on dialysis. Heart Vessels 2013; 28: 473–479. 22914904 10.1007/s00380-012-0273-2

[8] Hasegawa M, Ishii J, Kitagawa F, Takahashi K, Hayashi H, Koide S, Tomita M, Takahashi H, Ozaki Y, Yuzawa Y. Urinary neutrophil gelatinase-associated lipocalin as a predictor of cardiovascular events in patients with chronic kidney disease. Heart Vessels 2015; 30: 81–88. 24378882 10.1007/s00380-013-0454-7

[9] Mahmood SS, Wang TJ. The epidemiology of congestive heart failure: the Framingham Heart Study perspective. Glob Heart 2013; 8: 77–82. 23998000 10.1016/j.gheart.2012.12.006 PMC3756692

[10] Tsutsui H, Isobe M, Ito H, et al. JCS 2017/JHFS 2017 Guideline on Diagnosis and Treatment of Acute and Chronic Heart Failure—Digest Version. Circ J 2019; 83: 2084–2184. 31511439 10.1253/circj.CJ-19-0342

[11] D’Agostino RB, Sr, Grundy S, Sullivan LM, Wilson P. Validation of the Framingham coronary heart disease prediction scores: results of a multiple ethnic groups investigation. JAMA 2001; 286: 180–187. 11448281 10.1001/jama.286.2.180

[12] Nishimura K, Okamura T, Watanabe M, Nakai M, Takegami M, Higashiyama A, Kokubo Y, Okayama A, Miyamoto Y. Predicting coronary heart disease using risk factor categories for a Japanese urban population, and comparison with the framingham risk score: the suita study. J Atheroscler Thromb 2014; 21: 784–798. 24671110 10.5551/jat.19356

[13] McCullough PA. Anemia of cardiorenal syndrome. Kidney Int Suppl (2011) 2021; 11: 35–45. 33777494 10.1016/j.kisu.2020.12.001 PMC7983020

[14] DeLong ER, DeLong DM, Clarke-Pearson DL. Comparing the areas under two or more correlated receiver operating characteristic curves: a nonparametric approach. Biometrics 1988; 44: 837–845. 3203132

[15] Zheng Y, Parast L, Cai T, Brown M. Evaluating incremental values from new predictors with net reclassification improvement in survival analysis. Lifetime Data Anal 2013; 19: 350–370. 23254468 10.1007/s10985-012-9239-z PMC3686882

[16] Niizuma S, Iwanaga Y, Yahata T, Tamaki Y, Goto Y, Nakahama H, Miyazaki S. Impact of left ventricular end-diastolic wall stress on plasma B-type natriuretic peptide in heart failure with chronic kidney disease and end-stage renal disease. Clin Chem 2009; 55: 1347–1353. 19460838 10.1373/clinchem.2008.121236

[17] McCullough PA, Duc P, Omland T, et al. B-type natriuretic peptide and renal function in the diagnosis of heart failure: an analysis from the Breathing Not Properly Multinational Study. Am J Kidney Dis 2003; 41: 571–579. 12612980 10.1053/ajkd.2003.50118

[18] Kremers B, Wübbeke L, Mees B, Ten Cate H, Spronk H, Ten Cate-Hoek A. Plasma Biomarkers to Predict Cardiovascular Outcome in Patients With Peripheral Artery Disease: A Systematic Review and Meta-Analysis. Arterioscler Thromb Vasc Biol 2020; 40: 2018–2032. 32640905 10.1161/ATVBAHA.120.314774 PMC7447177

[19] Takase H, Dohi Y. Kidney function crucially affects B-type natriuretic peptide (BNP), N-terminal proBNP and their relationship. Eur J Clin Invest 2014; 44: 303–308. 24372567 10.1111/eci.12234

[20] Palazzuoli A, Ruocco G, Pellegrini M, Martini S, Del Castillo G, Beltrami M, Franci B, Lucani B, Nuti R. Patients with cardiorenal syndrome revealed increased neurohormonal activity, tubular and myocardial damage compared to heart failure patients with preserved renal function. Cardiorenal Med 2014; 4: 257–268. 25737690 10.1159/000368375 PMC4299262

[21] Sarnak MJ, Levey AS, Schoolwerth AC, Coresh J, Culleton B, Hamm LL, McCullough PA, Kasiske BL, Kelepouris E, Klag MJ, Parfrey P, Pfeffer M, Raij L, Spinosa DJ, Wilson PW. Kidney disease as a risk factor for development of cardiovascular disease: a statement from the American Heart Association Councils on Kidney in Cardiovascular Disease, High Blood Pressure Research, Clinical Cardiology, and Epidemiology and Prevention. Circulation 2003; 108: 2154–2169. 14581387 10.1161/01.CIR.0000095676.90936.80

[22] McLellan J, Bankhead CR, Oke JL, Hobbs FDR, Taylor CJ, Perera R. Natriuretic peptide-guided treatment for heart failure: a systematic review and meta-analysis. BMJ Evid Based Med 2020; 25: 33–37. 10.1136/bmjebm-2019-111208 PMC7029248 31326896

[23] Heerspink HJ, Kröpelin TF, Hoekman J, de Zeeuw D. Drug-Induced Reduction in Albuminuria Is Associated with Subsequent Renoprotection: A Meta-Analysis. J Am Soc Nephrol 2015; 26: 2055–2064. 25421558 10.1681/ASN.2014070688 PMC4520175

[24] Xie X, Liu Y, Perkovic V, Li X, Ninomiya T, Hou W, Zhao N, Liu L, Lv J, Zhang H, Wang H. Renin-Angiotensin System Inhibitors and Kidney and Cardiovascular Outcomes in Patients With CKD: A Bayesian Network Meta-analysis of Randomized Clinical Trials. Am J Kidney Dis 2016; 67: 728–741. 26597926 10.1053/j.ajkd.2015.10.011

[25] Chun KJ, Jung HH. SGLT2 Inhibitors and Kidney and Cardiac Outcomes According to Estimated GFR and Albuminuria Levels: A Meta-analysis of Randomized Controlled Trials. Kidney Med 2021; 3: 732–744.e1. 34746739 10.1016/j.xkme.2021.04.009 PMC8551546

[26] Furukawa S, Sakai T, Niiya T, Miyaoka H, Miyake T, Yamamoto S, Tanaka K, Ueda T, Senba H, Torisu M, Minami H, Matsuura B, Hiasa Y, Miyake Y. B-type natriuretic peptide and renal function in Japanese patients with type 2 diabetes mellitus: The Dogo Study. Endocr J 2017; 64: 1131–1136. 28855437 10.1507/endocrj.EJ17-0256

[27] Naganuma T, Takemoto Y, Taiyou O, Hirokazu T, Masaki M, Maeda S, Nakatani T. Risk of cardiovascular disease in kidney donors as a chronic kidney disease cohort. Mol Med Rep 2012; 5: 7–11. 21993905 10.3892/mmr.2011.629

[28] Wang TJ, Gona P, Larson MG, Tofler GH, Levy D, Newton-Cheh C, Jacques PF, Rifai N, Selhub J, Robins SJ, Benjamin EJ, D'Agostino RB, Vasan RS. Multiple biomarkers for the prediction of first major cardiovascular events and death. N Engl J Med 2006; 355: 2631–2639. 17182988 10.1056/NEJMoa055373

